# Probable Creutzfeldt-Jakob Disease With Negative Cerebrospinal Fluid (CSF) 14-3-3 Protein: Diagnostic Value of Diffusion-Weighted MRI

**DOI:** 10.7759/cureus.108879

**Published:** 2026-05-15

**Authors:** El Mahdi Choukri, Abdelilah Bouymajjan, Zakaria Boulahcen, Chetoui Ayoub, Sara Omari Tadlaoui, Siham Alaoui Rachidi

**Affiliations:** 1 Diagnostic and Interventional Radiology, Mohammed VI University Hospital, Tangier, MAR; 2 Radiology, Faculty of Medicine and Pharmacy, Abdelmalek Essaâdi University, Tangier, MAR

**Keywords:** 14-3-3 protein, basal ganglia, cortical ribboning, creutzfeldt-jakob disease, diffusion-weighted imaging, myoclonus, prion disease, rapidly progressive neuropsychiatric deterioration

## Abstract

Creutzfeldt-Jakob disease is a rare prion disorder that may present with rapidly progressive neurological and neuropsychiatric deterioration, making early diagnosis challenging. We report the case of a 55-year-old woman admitted for rapidly progressive balance impairment evolving over three months, associated with dysarthria, upper-limb dystonia, visual and auditory hallucinations, progressive loss of autonomy, mutism, refusal of oral intake, and myoclonus involving the face and limbs. A history of a similar neurological disorder in her mother was reported. Routine cerebrospinal fluid (CSF) analysis was unremarkable, onconeural antibody testing was negative, and cerebrospinal fluid 14-3-3 protein was negative. Electroencephalography showed slowing with short pseudo-periodic activity over the right hemisphere. Repeat brain magnetic resonance imaging demonstrated bilateral asymmetric cortical diffusion restriction, more marked in the right frontoparietotemporal region and the left parieto-occipital cortex, associated with the involvement of the right caudate and lentiform nuclei and the left caudate nucleus. There was no pathological enhancement, hemorrhage, or thalamic involvement.

Based on the rapidly progressive neurological course, myoclonus, extrapyramidal signs, suggestive electroencephalographic abnormalities, and characteristic cortical-basal ganglia diffusion restriction, a diagnosis of probable Creutzfeldt-Jakob disease was favored. This case highlights the diagnostic value of diffusion-weighted magnetic resonance imaging in suspected prion disease, especially when cerebrospinal fluid 14-3-3 protein is negative, and emphasizes the importance of considering a possible familial background when a similar disorder is reported in a first-degree relative.

## Introduction

Creutzfeldt-Jakob disease is a rare prion disorder characterized by rapidly progressive neurological deterioration and poor prognosis [1-3]. Its clinical presentation is heterogeneous and may include cognitive or behavioral changes, cerebellar dysfunction, visual symptoms, pyramidal or extrapyramidal signs, myoclonus, and akinetic mutism [1,3]. Because these manifestations overlap with several potentially treatable mimics, including autoimmune, infectious, metabolic, toxic, and paraneoplastic encephalopathies, early diagnosis remains challenging [3,4].

Brain magnetic resonance imaging has become a major supportive tool in suspected Creutzfeldt-Jakob disease, particularly when diffusion-weighted imaging demonstrates cortical ribboning and/or basal ganglia signal abnormalities [4-7]. Typical supportive MRI findings include restricted diffusion or hyperintensity involving the cerebral cortex, caudate nucleus, and putamen, often better appreciated on diffusion-weighted imaging than on conventional sequences [4-7]. Electroencephalography and cerebrospinal fluid biomarkers can further support the diagnosis; however, negative cerebrospinal fluid 14-3-3 protein does not exclude Creutzfeldt-Jakob disease [3,8,9].

We report the case of a 55-year-old woman with rapidly progressive neurological deterioration, negative cerebrospinal fluid 14-3-3 protein, suggestive electroencephalographic abnormalities, and characteristic cortical-basal ganglia diffusion restriction on repeat brain MRI. This case emphasizes the central role of diffusion-weighted imaging in the diagnostic approach to suspected prion disease, particularly when cerebrospinal fluid 14-3-3 protein is negative and when a possible familial background is reported.

## Case presentation

A 55-year-old woman with an unremarkable personal medical history was admitted for rapidly progressive neurological deterioration. Her family history was notable for a reportedly similar neurological disorder in her deceased mother. The patient initially presented with balance impairment evolving over approximately two months, associated with dysarthria, dystonia of the left upper limb, and visual and auditory hallucinations. During the following month, her condition worsened markedly, with mutism, loss of autonomy, bilateral upper-limb dystonia, refusal of oral intake, and myoclonus involving the face and limbs.

On clinical examination, the patient was hemodynamically stable with no respiratory distress. Mental status examination showed mutism and poor cooperation. Cranial nerve examination revealed facial palsy without oculomotor palsy. Motor examination was limited by poor cooperation, although no clear focal motor deficit was identified. Muscle tone was increased in the right upper limb, while no obvious tone abnormality was noted in the remaining limbs. Deep tendon reflexes were present and symmetric in all four limbs, and plantar responses were flexor bilaterally. Extrapyramidal features included upper-limb dystonia and myoclonus involving the face and limbs. Cerebellar testing could not be reliably performed because of poor cooperation. Pain sensation was preserved, but detailed sensory testing was limited. Gait assessment showed that standing and walking were impossible.

The overall clinical picture suggested rapidly progressive neuropsychiatric deterioration. Initial brain MRI performed during the previous hospitalization did not show specific abnormalities. Electroencephalography demonstrated background slowing with short runs of pseudo-periodic activity over the right hemisphere. Routine cerebrospinal fluid analysis was normal. Onconeural antibodies were negative, and cerebrospinal fluid 14-3-3 protein was negative. Laboratory tests, including complete blood count, ferritin, glycated hemoglobin, renal and liver function tests, thyroid-stimulating hormone, electrolytes, folate, and vitamin B12 levels, were unremarkable.

Because of the rapidly progressive clinical course and the absence of a clear diagnosis on the initial workup, repeat brain MRI was performed on a 1.5-T scanner. The protocol included diffusion-weighted imaging with apparent diffusion coefficient (ADC) mapping, axial T2 Propeller, sagittal T2 fluid-attenuated inversion recovery (FLAIR) fat-suppressed imaging, axial enhanced susceptibility-weighted angiography (eSWAN), three-dimensional T1-weighted imaging, and post-gadolinium T1-weighted sequences.

**Figure 1:**
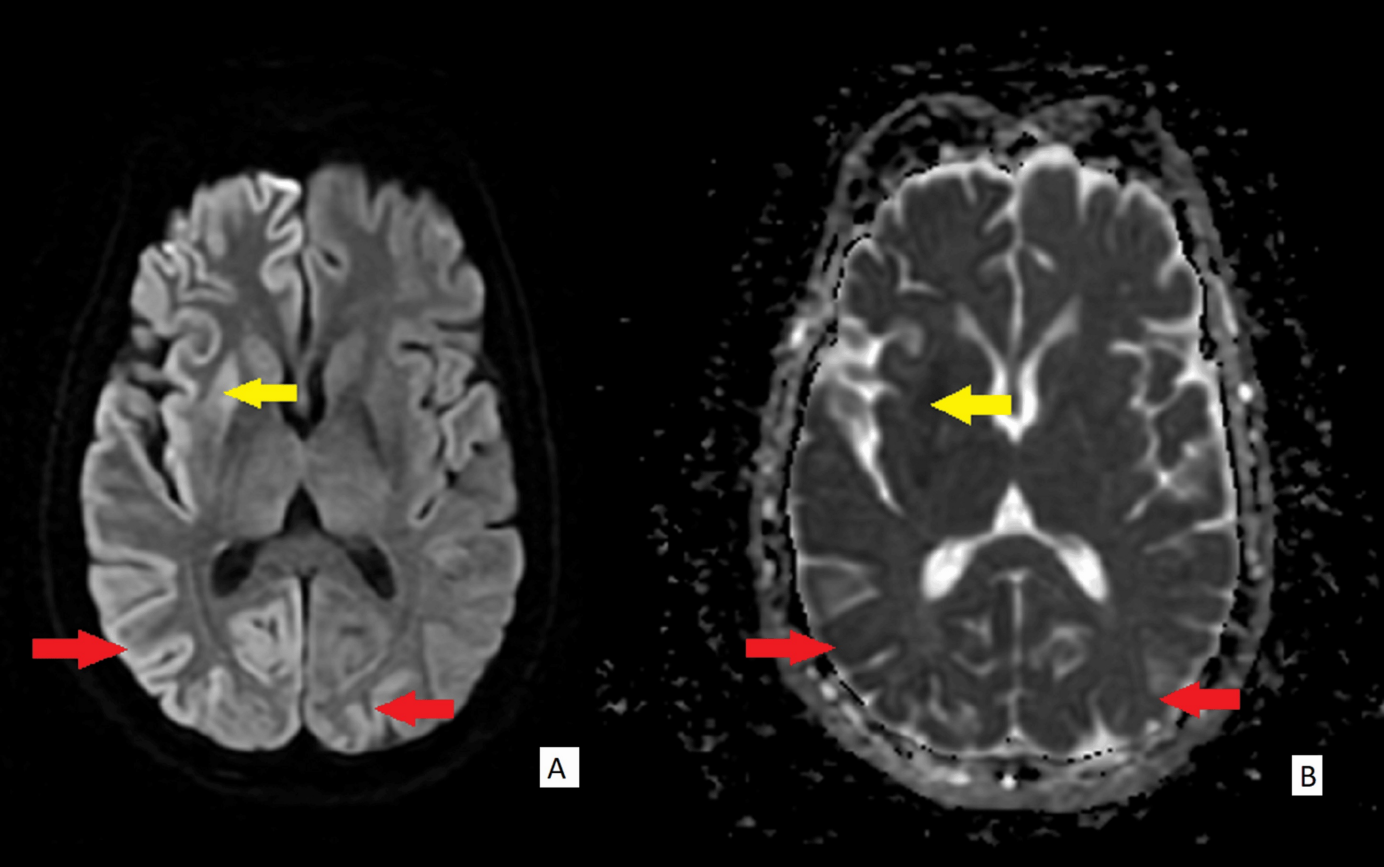
Diffusion-weighted imaging and ADC map Diffusion-weighted image (A) and corresponding apparent diffusion coefficient (ADC) map (B) showing bilateral asymmetric cortical diffusion restriction, more marked in the right frontoparietotemporal cortex and the left parieto-occipital cortex. The involved cortical regions show high signal on diffusion-weighted imaging with corresponding low ADC values, consistent with true diffusion restriction. Red arrows indicate bilateral cortical diffusion restriction, and yellow arrows indicate basal ganglia involvement.

Diffusion-weighted imaging demonstrated bilateral asymmetric cortical hyperintensity with corresponding low ADC values, consistent with true diffusion restriction. The abnormalities were more marked in the right frontoparietotemporal cortex and the left parieto-occipital cortex (Figure 1). Diffusion restriction also involved the right caudate and lentiform nuclei and the left caudate nucleus, while the thalami were spared.

**Figure 2:**
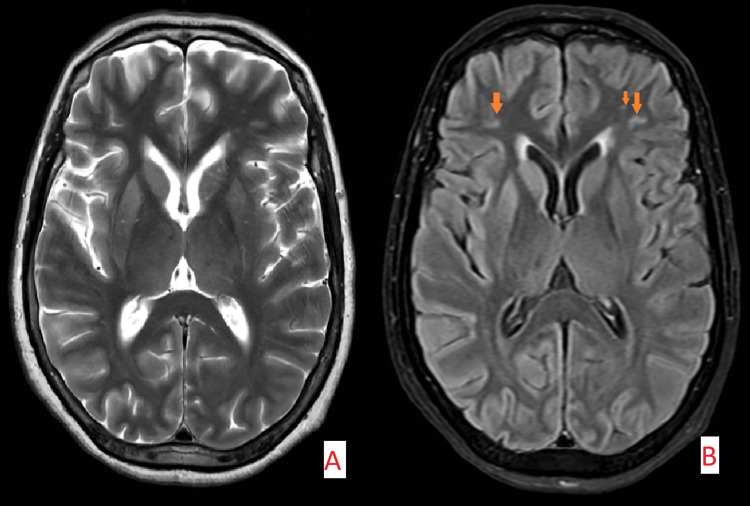
T2 and FLAIR sequences T2-weighted image (A) and FLAIR image (B) showing chronic microangiopathic white matter changes corresponding to Fazekas grade 2. Subtle cortical signal abnormality in the involved regions is better appreciated in correlation with diffusion-weighted imaging. Orange arrows indicate chronic microangiopathic white matter hyperintensities. FLAIR: Fluid-attenuated inversion recovery.

On T2 and FLAIR sequences, subtle cortical hyperintensity was observed in the involved regions. The associated chronic microangiopathic white matter changes were also present, corresponding to Fazekas grade 2 (Figure 2).

**Figure 3:**
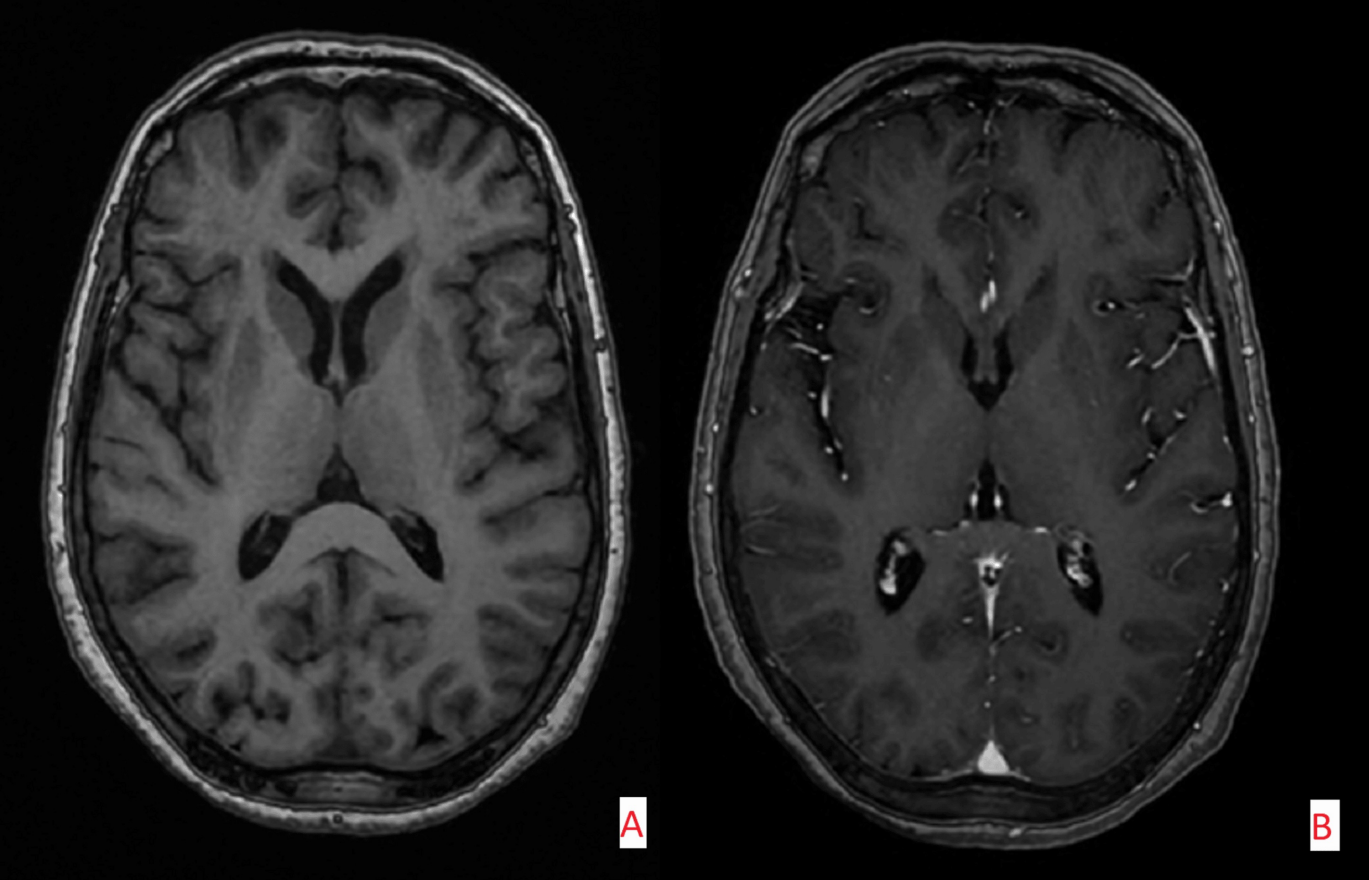
Pre- and post-contrast T1-weighted sequences Pre-contrast T1-weighted image (A) and post-gadolinium T1-weighted image (B) showing no mass-like lesion, no significant mass effect, and no abnormal enhancement.

Three-dimensional T1-weighted imaging showed no mass-like lesion or significant mass effect. Post-contrast T1-weighted sequences demonstrated no abnormal enhancement (Figure 3).

The eSWAN sequence showed no intracranial hemorrhage or abnormal susceptibility signal. Additional incidental, non-specific findings included partial empty sella and mild tortuosity of the optic nerves with slight distension of the optic nerve sheaths.

Given the rapidly progressive neurological decline, myoclonus, extrapyramidal signs, suggestive electroencephalographic abnormalities, negative available first-line workup, and characteristic cortical-basal ganglia diffusion restriction on repeat MRI, a diagnosis of probable Creutzfeldt-Jakob disease was favored. Autoimmune encephalitis remained an important potentially treatable mimic; therefore, additional investigations, including an autoimmune encephalitis antibody panel, total tau protein testing, and thoracoabdominopelvic computed tomography, were planned.

Because autoimmune encephalitis remained a potentially treatable differential diagnosis, empirical immunotherapy was initiated while additional investigations were pending. Intravenous immunoglobulin was administered at a dose of 28 g/day for five days. The available medical record did not document whether corticosteroids were specifically considered or the reason why corticosteroids were not administered. The patient was subsequently discharged. Post-discharge follow-up was not documented in the available medical record; therefore, subsequent clinical evolution and final results of the planned investigations could not be assessed.

## Discussion

Creutzfeldt-Jakob disease is a rare and fatal prion disorder that typically presents with rapidly progressive neurological decline, often associated with myoclonus, cerebellar dysfunction, pyramidal or extrapyramidal signs, behavioral changes, visual symptoms, and akinetic mutism [1-3]. The diagnosis can be difficult in the early stage because several potentially treatable disorders may mimic its presentation, including autoimmune encephalitis, infectious encephalitis, metabolic or toxic encephalopathies, and paraneoplastic syndromes [3,4].

According to revised diagnostic criteria for probable Creutzfeldt-Jakob disease [2,3], the diagnosis may be supported by rapidly progressive dementia or neuropsychiatric deterioration associated with at least two typical clinical features, such as myoclonus, visual or cerebellar signs, pyramidal or extrapyramidal signs, or akinetic mutism, together with at least one supportive investigation, including characteristic MRI abnormalities, typical electroencephalographic findings, or positive cerebrospinal fluid biomarkers. In the present case, the patient fulfilled the clinical-radiological pathway for probable Creutzfeldt-Jakob disease, given the rapidly progressive neurological decline, myoclonus, extrapyramidal signs, akinetic mutism, suggestive electroencephalographic abnormalities, and characteristic diffusion restriction involving multiple cortical regions and the basal ganglia. However, because of the reported similar neurological disorder in her mother and the absence of prion protein gene (PRNP) genetic testing, a genetic prion disease could not be excluded; therefore, the diagnosis was cautiously framed as probable Creutzfeldt-Jakob disease rather than definitively sporadic disease.

In the present case, the clinical course was highly suggestive of rapidly progressive neuropsychiatric deterioration, with balance impairment, dysarthria, hallucinations, progressive mutism, loss of autonomy, dystonia, and myoclonus. Although the initial MRI was reported as non-specific, the repeat MRI demonstrated a characteristic pattern of bilateral asymmetric cortical diffusion restriction, associated with basal ganglia involvement. This evolution highlights the importance of repeating MRI when clinical suspicion remains high and the initial imaging does not explain the neurological deterioration.

Diffusion-weighted imaging (DWI) is one of the most useful MRI sequences in suspected Creutzfeldt-Jakob disease. Typical findings include cortical ribboning and/or signal abnormalities involving the caudate nucleus and putamen, often more conspicuous on DWI and ADC maps than on conventional sequences [4-7]. In our patient, true diffusion restriction was supported by high DWI signal with corresponding low ADC values, involving the right frontoparietotemporal cortex, the left parieto-occipital cortex, the right caudate and lentiform nuclei, and the left caudate nucleus. The absence of pathological enhancement, hemorrhage, mass effect, or thalamic involvement helped narrow the radiological differential diagnosis.

The negative cerebrospinal fluid 14-3-3 protein result did not exclude Creutzfeldt-Jakob disease. CSF 14-3-3 is a marker of rapid neuronal injury rather than a disease-specific marker, and its diagnostic performance is imperfect [8]. Therefore, interpretation must rely on the full clinical-radiological context rather than on a single biomarker. In this case, the combination of rapidly progressive neurological decline, myoclonus, extrapyramidal signs, suggestive EEG abnormalities, and characteristic cortical-basal ganglia diffusion restriction supported the diagnosis of probable Creutzfeldt-Jakob disease.

Electroencephalography may provide supportive evidence, particularly when periodic or pseudo-periodic abnormalities are present, but it is not sufficient alone to establish the diagnosis [9]. In our patient, EEG showed slowing with short runs of pseudo-periodic activity over the right hemisphere, which was considered supportive in the appropriate clinical context. Similarly, the family history of a reportedly similar neurological disorder in the patient’s mother raised the possibility of a familial background. However, in the absence of PRNP genetic testing or neuropathological confirmation, a genetic prion disease could not be confirmed [10].

The main limitation of this case is the absence of additional diagnostic testing, including real-time quaking-induced conversion (RT-QuIC), PRNP genetic analysis, and neuropathological confirmation. RT-QuIC was not performed. This is particularly relevant because RT-QuIC is currently among the most specific premortem diagnostic tests for Creutzfeldt-Jakob disease [3]. The reason for the absence of RT-QuIC testing was not documented in the available medical record; possible contributing factors may include local availability or send-out testing limitations. PRNP genetic testing was not performed, limiting the ability to distinguish sporadic from genetic prion disease, especially given the reported history of a similar neurological disorder in the patient’s mother. Therefore, the diagnosis should remain framed as probable Creutzfeldt-Jakob disease. Additional limitations include the absence of documented follow-up after discharge and the lack of final results for the planned autoimmune encephalitis panel, total tau protein testing, and thoracoabdominopelvic computed tomography. Despite these limitations, the case remains valuable because it illustrates the central diagnostic role of repeat diffusion-weighted MRI in a patient with rapidly progressive neuropsychiatric deterioration and negative CSF 14-3-3 protein.

## Conclusions

Creutzfeldt-Jakob disease should be considered in patients presenting with rapidly progressive neurological deterioration, especially when myoclonus, extrapyramidal signs, akinetic mutism, and suggestive electroencephalographic abnormalities are present. This case illustrates the diagnostic value of repeat brain MRI, particularly diffusion-weighted imaging and ADC mapping, in demonstrating characteristic cortical and basal ganglia diffusion restriction even when cerebrospinal fluid 14-3-3 protein is negative. Although confirmatory testing was not available, the clinical, electroencephalographic, and radiological findings supported a diagnosis of probable Creutzfeldt-Jakob disease. The reported history of a similar neurological disorder in a first-degree relative also highlights the importance of considering a possible familial background and the potential role of genetic testing when available.

## References

[1] Geschwind MD (2015). Prion diseases. Continuum (Minneap Minn).

[2] Zerr I, Kallenberg K, Summers DM (2009). Updated clinical diagnostic criteria for sporadic Creutzfeldt-Jakob disease. Brain.

[3] Hermann P, Appleby B, Brandel JP (2021). Biomarkers and diagnostic guidelines for sporadic Creutzfeldt-Jakob disease. Lancet Neurol.

[4] Fragoso DC, Gonçalves Filho AL, Pacheco FT (2017). Imaging of Creutzfeldt-Jakob disease: imaging patterns and their differential diagnosis. Radiographics.

[5] Vitali P, Maccagnano E, Caverzasi E (2011). Diffusion-weighted MRI hyperintensity patterns differentiate CJD from other rapid dementias. Neurology.

[6] Shiga Y, Miyazawa K, Sato S (2004). Diffusion-weighted MRI abnormalities as an early diagnostic marker for Creutzfeldt-Jakob disease. Neurology.

[7] Collie DA, Sellar RJ, Zeidler M, Colchester AC, Knight R, Will RG (2001). MRI of Creutzfeldt-Jakob disease: imaging features and recommended MRI protocol. Clin Radiol.

[8] Muayqil T, Gronseth G, Camicioli R (2012). Evidence-based guideline: diagnostic accuracy of CSF 14-3-3 protein in sporadic Creutzfeldt-Jakob disease: report of the guideline development subcommittee of the American Academy of Neurology. Neurology.

[9] Steinhoff BJ, Zerr I, Glatting M, Schulz-Schaeffer W, Poser S, Kretzschmar HA (2004). Diagnostic value of periodic complexes in Creutzfeldt-Jakob disease. Ann Neurol.

[10] Kovács GG, Puopolo M, Ladogana A (2005). Genetic prion disease: the EUROCJD experience. Hum Genet.

